# Nanomotors for Nucleic Acid, Proteins, Pollutants and Cells Detection

**DOI:** 10.3390/ijms19061579

**Published:** 2018-05-25

**Authors:** Alejandro Baeza, María Vallet-Regí

**Affiliations:** 1 Department of Chemistry in Pharmaceutical Sciences, School of Pharmacy, Universidad Complutense de Madrid, Instituto de Investigación Sanitaria Hospital 12 de Octubre i+12, Plaza Ramón y Cajal s/n, 28040 Madrid, Spain; 2 Networking Research Center on Bioengineering, Biomaterials and Nanomedicine (CIBER-BBN), Av. Monforte de Lemos, 3-5. Pabellón 11, 28029 Madrid, Spain

**Keywords:** nanomotors, biomolecule detection, self-propelled nanosensors

## Abstract

The development of nanomachines able to operate at the nanoscale, performing complex tasks such as drug delivery, precision surgery, or cell detection, constitutes one of the most important challenges in nanotechnology. The principles that rule the nanoscale are completely different from the ones which govern the macroscopic world and, therefore, the collaboration of scientists with expertise in different fields is required for the effective fabrication of these tiny machines. In this review, the most recent advances carried out in the synthesis and application of nanomachines for diagnosis applications will be presented in order to provide a picture of their potential in the detection of important biomolecules or pathogens in a selective and controlled manner.

## 1. Introduction

**Figure 1:**
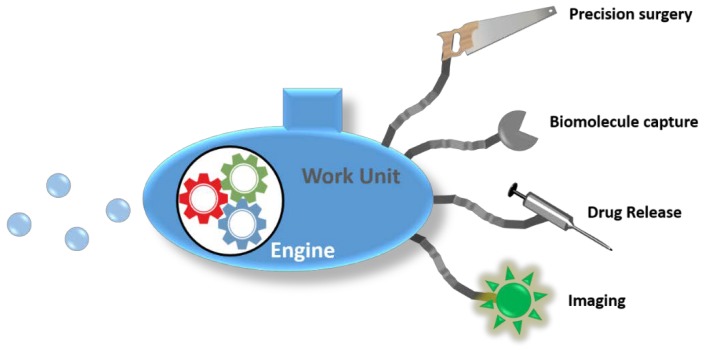
Principles of nanomotor for sensing: engine and sensors.

The development of tiny nanomotors able to precisely perform complex surgical operations inside a patient, to administrate drugs directly in the diseased tissue, or to analyze the amount of pathogenic agents with astonishing accuracy is no longer a science fiction tale. Since the inspiring lecture of Prof. Feynman in 1959 about the unlimited possibilities within the nanoscale [1], scientists of many different fields have joined forces in order to develop novel technologies capable of observing matter in atomic scale, to manipulate atoms or molecules with extraordinary precision and to assembly nanometric objects into autonomous nanomachines. A nanomotor should be composed of at least two principal parts: an engine and a work unit. The engine is responsible for the production of kinetic energy from a certain potential energy source such as chemical energy, radiations, or magnetic fields, among others. This engine provides motion capacity to the nanodevice. The work unit should be able to recognize a signal, which can be a biomolecule, and then—to provide a controlled response such as radiation emission—open or close valves or to provoke a directional change in the translational motion of the device (Figure 1). The principles which govern the fabrication of macroscopic machines are not valid for the construction of nanometric ones. Locomotion in an environment with a low Reynolds number is strongly affected by Brownian motion and, therefore, the physical principles which rule the nanomotor’s behavior in this scale should be taken into account [2,3,4]. There are different strategies available for achieving locomotion at the nanoscale depending on the fuel or energy employed. Nanomotors powered by chemical reactions usually create reagents/products or electrical gradients which propel the systems by self-difussiophoresis [5] or self-electrophoresis [6] mechanisms, or produce oxygen bubbles which propel the nanodevice in a similar way to a conventional rocket [7,8]. Magnetic fields have also been employed for the propulsion of nanomotors. In this case, helical or flexible microwires are able to exhibit fast locomotion when they are exposed to rapid changes in the magnetic field orientation which mimicks bacterial flagella [9,10]. The exposure of the material surface of a nanomotor to certain light wavelengths catalyzes reactions producing chemical gradients which can be used as propulsion force. Thus, TiO_2_ [11] or AgCl nanoparticles have shown excellent locomotion capacities under UV exposure, being able to destroy pollutants or bacteria at the same time [12]. Finally, the use of ultrasounds (US) for propelling nanomotors has provided an interesting alternative in order to guide them to specific locations, even within living organisms, due to the capacity to focus these mechanical waves with high precision [13].

Despite the fascinating nature of these tiny motors, which are able to move from one point to another, only locomotion capacity is not quite useful, being necessary to provide to the motors the capacity to carry out useful tasks such as drug release, biomolecule sensing, or precise nanosurgery, among others. In recent years, nanomotor scientists have struggled with the unique properties of the nanoscale in order to engineer smart systems capable of performing complex tasks such as capturing and transporting nanometric cargoes, releasing therapeutic agents, removing pollutants, carrying out delicate surgeries, or capturing and analyzing important biomolecules, among others [14]. The earliest nanomotors were designed in order to capture polymeric nanoparticles by electrostatic interactions or by the formation of biotin–streptavidin bridges, as a proof of concept of the ability to transport cargo to different locations [15]. Later, the capacity to transport and release nanoparticles was applied for the transportation of drug loaded nanocarriers in order to lead them close to the diseased cells. As a few examples of drug delivery applications, Wang et al. have described a magnetically propelled nanowire able to transport polymeric nanoparticles, loaded with a potent cytotoxic compound, doxorubicin (Dox), or iron encapsulated liposomes. These nanomotors navigated into a microfluidic channel controlled by an external magnetic field and were able to release the cargo on demand [16]. The control of the performance of these nanowires is so precise that it was even possible to release the transported nanoparticle in the surroundings of tumoral cells, which could be useful for the future application of these nanomachines in the fight against complex diseases, such as cancer [17]. Engineered polymeric tubular microrockets have shown capacity to release Dox into tumoral cells under ultrasounds application [18]. Water detoxification is another interesting task which can be addressed with nanomotors. The motile behavior of these systems enables the achievement of a homogeneous distribution in the contaminated area, as well as allowing the motors to be driven to the desired place. Moreover, motor motion facilitates the pollutants capture improving their efficacy, in comparison with static systems [19]. The possibility of precisely controlling the motion of these nanometric machines has opened a new way to perform delicate surgeries in order to minimize the side damages caused by surgery and to accelerate the patient recovery. Thus, Pane et al. have developed micromotors able to navigate within the eye, perform intraocular surgery, and even deliver drugs in the zone [20]. Clinical diagnosis is one of the field that has benefited most from the use of nanomotors, receiving great attention in recent years. The use of self-propelled nanodevices capable of capturing “on-the fly” specific analytes from complex samples such as blood or urine, and to deliver them into clean detection spots, allows the detection of important biomolecules without tedious purification steps, and without the need to concentrate the sample. Thus, these self-propelled nanomotors can carry out three analytical steps at once: capture, isolation, and detection, saving a considerable amount of time. Additionally, it is not necessary to design complex microfluidic systems due to the autonomous and directed motion capacity of the motors and therefore, these systems can be easily integrated within simple and portable kits for the detection of important biomolecules facilitating the diagnosis of many different pathologies. In this review, some of the most representative advances of the use of nanomotors for biomolecule and cell detection carried out in recent years will be presented. The aim of this review is not to present a comprehensive description of the current state of the art, but to describe a few representative examples of the application of nanomotors in the detection of important biomolecules such as proteins or DNA/RNA strands and cells, in order to provide a picture of the potential of this technology in the diagnosis field.

## 2. Nanomotors for Protein Detection

Proteins are probably the most important biomolecules for life. They perform practically all the key tasks in living systems, from structural to metabolic and sensing functions, being ubiquitous in all organisms. There are many pathologies such as cancer, neurological disorders, or cardiovascular diseases, among others, which are characterized by an unbalanced production of proteins or by the production of mutated or altered proteins, which are not present in the healthy organism. Therefore, the development of precise and rapid analytical methods for their detection is of paramount importance for the early diagnosis of these diseases. Detection of proteins is not a simple process, especially when they are existent in the sample in a very low amount. This process usually requires the application of tedious purification steps, which consume a large amount of time and significantly increase the cost of the analysis. As mentioned above, the use of nanomotors able to specifically capture proteins from an untreated complex sample, and transport them into a clean spot for subsequent detection, can significantly facilitate the process, allowing a rapid detection of the desired protein. 

**Figure 2:**
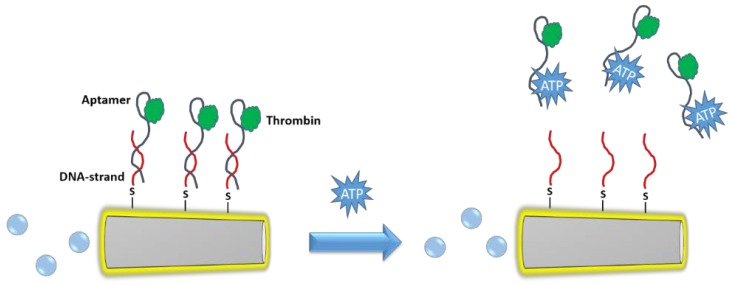
Microrocket for capture and release of thrombin mediated by aptamers and ATP.

Wang et al. have employed aptamers-modified microrockets for the capture of thrombin from human serum samples [21]. The system is based on metallic microtubes (microrockets) which present Pt in the inner surface and Au in the external face. The Pt core is responsible for the generation of the propulsion force, because this metal catalyzes the decomposition of hydrogen peroxide into water and molecular oxygen. Thus, when the motor is submerged in a solution which contains small amounts of hydrogen peroxide, the continuous bubble formation propels the rocket following a jet-based propulsion mechanism [22]. The gold surface was decorated with thiolated aptamers thanks to the high affinity of the sulfur atom by Au, which rapidly forms a covalent bond at room temperature in the aqueous phase. Aptamers are DNA strands which can bind specifically to different biomolecules through intermolecular interactions between the tridimensional structure of the DNA strand and specific points of the biomolecule [23]. Thus, the explicit sequence of the attached aptamer allowed the capture of thrombin from the sample and its transportation into a clean zone. Additionally, the nanomotor surface was decorated with a mixed binding aptamer which contained two recognition regions for ATP and thrombin, respectively. Thus, this system was able to capture thrombin and release it when ATP was present, as a consequence of a conformational change caused by the ATP binding event which triggered the aptamer release from the Au surface (Figure 2). The resulting nanomotor has demonstrated excellent properties for the detection of important biomolecules because this methodology can be easily tuned for the capture of many different biological moieties, thanks to the great versatility of aptamers. Antibodies can also be attached to the surface of tubular microrockets in order to capture biomolecules. García et al. have immobilized antibodies against immunoglobulin G (Anti-IgG) on the polymeric surface of Ni/Pt microtubes coated with poly(3,4-ethylenedioxythiophene) (PEDOT) functionalized with carboxylic acids [24]. The antibody was attached employing the well-known carbodiimide chemistry which activates the carboxylic acids, located on the polymer-coated microtube surface, transforming them into more reactive esters that react with the amine groups, naturally present in the antibody. Similar to the previous system, this nanomotor was capable of propelling itself in a media enriched in H_2_O_2_ by oxygen bubble formation, capturing the protein during flying. Thanks to the presence within the tube of a Ni core, which interact with magnetic fields, this system was magnetically guided throughout a microchip channel network, allowing the transportation of the captured proteins towards clean spots.

The same methodology was reported for the detection of multiple analytes using microrockets functionalized with several antibodies [25]. The antigens were attached on gold nanoparticles with different sizes and shapes which act as tags and, therefore, the antigen capture process could be multiplexed detected by direct microscopic observation of the micromotor-microscopic tag movement. The authors have also demonstrated that the rocket’s own motion facilitates the mixing of the media enhancing the rate of the protein capture. One important advantage of this technology is its easy adaptability which can allow the detection of a vast number of biomolecules and the use of multiple tags, that permit a rapid detection by different spectroscopic or fluorescence methods. Wu et al. have reported the use of polyaniline/Pt microtubes for the detection of cancer biomarkers [26]. In this system, gold nanoparticles were sputtered on the microtube surface and a first antibody was then adsorbed on the gold surface in order to capture the cancer biomarker (anticarcinoembryonic antigen). A second antibody was conjugated on the surface of glycidyl methacrylate microspheres which binds to the microrocket which transports the biomarker, forming a sandwich complex and slowing their translation rate. It was possible to determine the amount of biomarkers present in a sample, measuring the motion rate change and counting the tags attached on the motor surface. This process allowed the determination of these biomarkers in 5 min, when the analyte was present in a concentration range of 1–1000 ng·mL^−1^. Wang et al. have developed graphene oxide (GO)-based microtubes which contains a platinum inner cavity, that catalyze the oxygen bubble formation, and are decorated with a ricin B aptamer tagged with fluorescein [27]. The aptamer was bound to the GO surface by π–π interactions between the nucleotide bases and the carbon surface. When ricin B was not present, the aptamer was adsorbed on the motor surface and the fluorescence was quenched by the close proximity between GO and fluorescein. The high affinity between ricin B toxin and the aptamer produced the aptamer release from the GO surface when this toxin was present in the media and, therefore, the fluorescence was recovered. This On–Off fluorescent response and the efficient mixing capacity of the micromotors allowed the rapid detection of this toxin without the need to isolate it from the original sample. Based on similar aptamer-functionalized GO microrockets, another research group has applied this type of system to the detection of mycotoxins in food samples [28]. The presence of mycotoxins such as ochratoxin A and fumonisine B1, which are produced by several fungi species that usually contaminate food samples, is related with many health problems in the animal and human feed. The use of this motors for their detection provides a rapid analysis with high sensitivity due to the improved mixing process cause by the motor motion and the excellent affinity of the aptamer by the toxins.

## 3. Nanomotors for Nucleic Acid Detection

**Figure 3:**
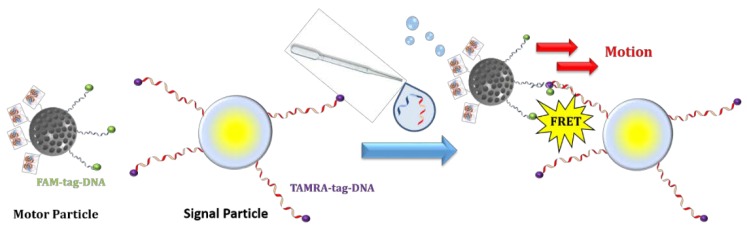
DNA motion-based assay based on Janus mesoporous silica nanoparticles.

Deoxyribonucleic acid (DNA) is one of the most important molecules in all living organisms on Earth. This molecule codes the entire information of an organism, being the instruction manual of each living being. DNA is copied to ribonucleic acid (RNA) in order to translate its coded information for protein synthesis. Therefore, the analysis of DNA or RNA is critical for the early diagnosis of a plethora of different diseases, from cardiovascular pathologies to cancer. Nowadays, the analysis of these important biopolymers is mainly based on polymerase chain reaction (PCR), which is a technique that mimics the natural DNA replication mechanism for amplifying the DNA strands present within a sample. This reaction increases the concentration of these macromolecules and facilitates their detection [29]. The main limitation of this, and other related techniques, is that they usually require time-consuming pre-purifications steps which increase the time of the analysis and also raises their cost. At this point, the use of nanomotors for DNA isolation and analysis can speed up the process due to the self-propelled behavior of these machines which can be employed in order to carry out the DNA isolation, similar to the case of the proteins mentioned above. In 2010, Wang et al. reported one of the first motor-based DNA sensors which exploited the observed speed differences in Au–Pt nanowires in the presence of silver [30]. In this assay, DNA strands complementary to the DNA analyte were attached on a gold surface. Then, a second DNA strand complementary to other section of the DNA analyte was attached on the surface of Ag nanoparticles. When the DNA analyte was present in the media, the Ag nanoparticles were retained on the surface by the formation of a sandwich DNA complex between the DNA attached on the gold surface, the DNA anchored on the Ag nanoparticle, and the DNA analyte, which acted as bridge. After this process, a solution with Au–Pt nanorods and H_2_O_2_ as fuel was added. The speed of the Au–Pt nanorods was strongly enhanced by the presence of Ag^+^ in the media and, therefore, the greater amount of DNA, the greater concentration of Ag^+^ in the media and the higher the nanomotor speed that was observed, it being possible to quantify the DNA concentration in the sample. Despite the innovative nature of this assay, its suitability was not very high because it required several steps. Additionally, the precise nanomotor speed determination is quite difficult. The same research group developed another motor-based DNA assay based on the use of tubular microrockets, asymmetrically covered with gold on one side and having a Pt core able to propel themselves in a solution of H_2_O_2_ [31]. In this system, thiolated DNA strands, which are complementary to certain DNA or RNA sequences present in pathogen organisms, were anchored on the gold surface of the micromotors. The analyte DNA or RNA sequences were tagged with fluorescent nanoparticles in order to facilitate their detection. These micromotors were capable of capturing the pathogen oligonucleotide strands “on-the-fly” and transporting them to a clean chamber, where the detection could be carried out without any interference. Minteer et al. have reported the development of other “on-the-fly” capture system but, in this case, the micromotor captured the engine instead of the signal if a certain DNA strand was present in the media [32]. This nanodevice was based on PEDOT/Au nanorods decorated on the gold surface with DNA strands which captured Pt nanoparticles functionalized with a second DNA strand. If the desired DNA was present in the environment, the nanorods captured the Pt nanoparticles (engines) by a sandwich hybridization between the three oligonucleotide strands causing the motion of the device. The average speed of these nanorods was dependent on the DNA amount and it indicated its concentration in the media. Another strategy also based on an indirect strategy to detect DNA was presented by Simmchen et al. employing Janus mesoporous silica nanoparticles [33]. In this case, the authors proposed the use of asymmetrically functionalized nanoparticles as nanomotors which were decorated with catalase (engine) in one hemisphere and DNA strands on the other side. Catalase was selected as engine because this enzyme catalyzes the decomposition of H_2_O_2_ generating oxygen bubbles with a high efficacy, significantly higher than Pt, allowing the motor motion in environments with a really low fuel concentration. These nanomotors were able to capture and transport a signal particle which were functionalized with DNA strands complementary to a certain DNA analyte sequence due to the formation of a sandwich complex, similar to the others mentioned above. In order to discard the possibility that the capture process could have occurred by unspecific interactions between both systems (the motor particle and the signal particle) fluorescein was attached at the end of the DNA attached on the motor particles and the DNA strands present on the signal particle were labeled with TAMRA. The assembly of both particles occasioned a clear fluorescence emission in the emission wavelength of TAMRA when the sample was irradiated in the excitation wavelength of fluorescein (Figure 3). The apparition of this phenomenon, called Föster resonance energy transfer (FRET), indicated that both fluorophores were placed closer than 5 nm, as a consequence of the DNA hybridization, corroborating that the motor-signal assembly was caused by the presence of the DNA analyte in the media. Moreover, employing a DNA analyte which has one base mismatched to the complementary strand, the FRET effect disappeared and the motion in the signal particles was not observed. This last property indicates the high specificity of this type of sensors.

The use of nanomotors for DNA or RNA detection is not exclusive for extracellular applications, as mentioned above, but these propelled systems can also be employed for real time detection of DNA or RNA inside cells [34]. This system was composed by a gold nanowire coated with graphene oxide (GO). A single-strand DNA sequence labeled with a fluorescent tag was adsorbed on the surface of the GO layer by π–π interactions, resulting in fluorescence quenching of the tag (Off state). This DNA sequence was carefully selected to be complementary with miRNA-21, which is overexpressed by many tumoral cells [35]. This nanomotor can be propelled by ultrasounds and it can be “injected” within a tumoral cell and, once there, the presence of miRNA-21 can detach the fluorescent DNA from the GO surface by specific sequence complementarity, resulting in fluorescent recovery within the cell (On state). Thus, the apparition of a fluorescent signal within the cells indicated that these cells were malignant. Keya et al. have recently published an interesting work in which the presence of certain DNA signals triggers the swarming behavior of kinesin-based nanomotors [36]. In this work, microtubules of tubulin (MT) were functionalized with one single DNA strand and tagged with a fluorophore in order to monitorize their motion by fluorescence microscopy. The propulsion force was provided by kinesins adhered on the surface of the MT which extract the energy from ATP. These micromotors were able to perform translational motion on the surface of a glass substrate without any interaction between them. However, if a certain DNA, which was partially complementary to the DNA located on the MT surface, was added to the media, the MT experienced aggregation, forming a swarm of around 100 MT each. Additionally, the authors incorporated an azobenzene molecule in each DNA strand in order to control the hybridization by the application of light. Azobenzene present *trans*–*cis* isomerization when UV or visible light is applied and this process alters the melting temperature of the hybridized DNA, being of 20 °C in the cis state and 60 °C in trans conformation. The application of UV light at 365 nm converted the azobenzene into the cis form, distorting the hybridization, and therefore MT performed motion in an individual state. If the sample was exposed to visible light at 480 nm, the azobenzene suffered isomerization to trans state, allowing the hybridization and triggering the swarming process. This process is completely reversible, it being possible to control the swarming behavior of these MT and their motion by the exposure to different light sources. 

## 4. Nanomotors for Pollutants and Small Molecules Detection

The analysis of pollutants in water, especially heavy metals, constitutes an important research area due to the increased contamination problems in industrialized society. One of the first motor-based assay of metals in water was reported by Wang et al. in 2009 [37]. This method exploited the dependence which exhibit the speed of Au-Pt nanorods with the presence of Ag^+^ in the media. The nanomotors that present a propulsion mechanism based on self-electrophoresis suffer a strong dependence with the presence of salts in the media, showing a linear speed decrease with the solution conductivity [38]. In contrast, the presence of silver produces a significant speed up to 5-fold showing a concentration dependence which was used for the determination of this element in water samples. The most plausible explanation of this acceleration is that Ag^+^ was adsorbed on the nanorods surface being reduced to Ag^0^ in the presence of H_2_O_2_, which produced changes in the catalytic power of these systems. This alteration in the motor speed caused by the presence of metal cations has also been employed for the determination of toxic metals in water samples. Pumera et al. have employed bubble-powered Pt micromotors for the determination of Pb^2+^ and Cd^2+^ [39]. These heavy metals poison the Pt surface, hampering the decomposition of H_2_O_2,_ and thus provoke a decrease in the speed of the nanomotor which was used for the concentration determination of these metals in the mM range. Micromotors can also be employed for pollutant removals instead of only as sensors. Sánchez et al. have employed tubular microjets with Pt in the inner space and coated with GO capable of capturing heavy metals, as Pb^2+^, due to the high adsorption of these metals on the oxygen moieties which are present on the carbon surface [40]. These microtubes contained a magnetic shell of Ni and Ni/Pt alloy in the middle, which allowed their magnetic guidance in order to control the motion of the motors and achieved the recovery and even the analysis of the captured lead. The application of these micromotors in contaminated water samples achieved a significant Pb^2+^ reduction from 1000 ppb to below 50 ppb, which pointed out the suitability of this type of system for water treatment. GO microrockets have been recently reported for the elimination of oil from water [41]. The application of these self-propelled nanodevices has also been proposed for the detection of chemical warfare agents (CWA). Wang et al. have reported the use of PEDOT/Au tubular microjets functionalized with catalase in the organic inner surface for the detection of sarin gas [42]. Catalase is inhibited by the presence of sarin gas and therefore, it is possible to detect the presence of this CWA through the observation of the motion decrease of these micromotors. H_2_O_2_ is the fuel of practically all the motors mentioned in this review but it is also possible to employ a nanomotor for its detection. For this purpose, Escarpa et al. have developed a motion-based assay of H_2_O_2_ in different matrices as mineral water, urine, plasma, or tumoral cell cultures [43]. This motor was based on microcones which released a surfactant (Sodium dodecyl sulfate) by the sharp edge which propelled the system by the difference in surface tension between both ends of the cone (Maragoni effect). The cone also released Horseradish peroxidase (HRP) which produced the oxidation of a colorless compound (TMBred) in the presence of H_2_O_2_ into the blue-colored oxidized species (TMBox). The concentration of H_2_O_2_ could be rapidly determined by UV-Vis spectroscopy because the cone’s own motion allowed the mixing of the reagents, speeding up the detection. Finally, other important biomolecules, such as sugars, have been determined using micromotors [44]. In this case, poly(3-aminophenylboronic acid) (PAPBA)/Ni/Pt microrocket was decorated with boronic acid on the external surface in order to capture sugars by the complexation reactions between the boronic acid and the hydroxyl groups of the monosaccharides.

## 5. Nanomotors for Cell Capture

**Figure 4:**
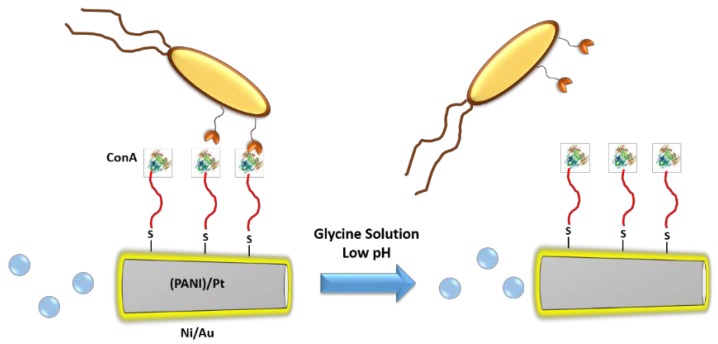
Bacteria capture and release by lectin-functionalized microrockets.

There are many diseases originated by living organism such as bacteria, fungi, and viruses, or which are caused by the pathological transformation of an organism’s own cells into malignant cells, as is the case with tumoral cells. The correct identification of these organisms is crucial for the appropriate diagnosis of the disease and the assignation of an effective treatment. This process usually requires the isolation of the pathogenic organism from the physiological fluids or tissues obtained from biopsies. These are very complex matrices and a plethora of different procedures as removal of the matrix components, isolation, and culture of the pathogen being and identification of the species by complex analytical methods, are required. For these reasons, the development of self-propelled nanomachines able to capture these organisms in a selective manner and then transport them to a different location in order to identify them could suppose a great advancement which could improve the diagnosis tools of the clinicians. Wang et al. have reported the use of lectin-functionalized microrockets for the capture and release of *Escherichia coli* (*E. coli*) [45]. Lectins are a family of proteins able to recognize many of the carbohydrate constituents of the bacteria membrane. In this work, the authors employed concanavalin A (ConA) which specifically binds to specific polysaccharides of Gram-negative bacteria as *E. coli*. The microrockets presented a size of around 8 µm and were composed of a polyaniline (PANI)/Pt bilayer core, for providing the propulsion capacity by decomposition of hydrogen peroxide, and a Ni/Au layers required for controlling the navigation, which was sputtered on one side of the motor. The gold surface was decorated with alkenethiol mixture of 6-mercaptohexanol and 11-mercatoundecanoic acid (MUA); the last contains a carboxylic group of the terminal end in order to provide the grafting points for the lectin attachment by carbodiimide chemistry. These microrockets were able to capture and transport *E. coli* selectively from a solution which contained either 5-fold excess of *Saccharomyces cerevisiae* (*S. cerevisiae*) or *Staphylococcus aureus* (*S. aureus*) which are pathogens responsible for urinary tract infections, because ConA does not present selectivity against the saccharides present in the membrane of these organisms. Additionally, the captured bacteria were effectively released using a glycine solution at low-pH because the complex lectin-polysaccharide was disrupted in these conditions (Figure 4). Finally, the nanomotors were able to capture and transport bacteria and lectin-functionalized poly d,l-lactic-co-glycolic acid (PLGA) microparticles loaded with magnetic iron oxide nanoparticles at the same time, which pointed out the possibility of using these systems as theranostic devices able to detect and treat bacterial infections.

In other work, the Wang’s group reported the fabrication of a three-segment Au@Ni@Au nanowire capable of capturing bacteria propelled by ultrasounds [46]. In this work, the nanowires were fabricated by template electrodeposition methods yielding Au@Ni@Au (0.8 μm/0.2 μm/0.8 μm) in which the Nickel segment was necessary for controlling the orientation of the nanowire by the application of a static magnetic field. The gold segments were decorated with MUA and then functionalized with recognition moieties (ConA or anti-protein A) able to recognize different bacteria such as *E. coli* or *S. aureus*, respectively. A concave end in the nanowire was introduced in the fabrication process employing a sacrificial Cu layer at the beginning of the electrodeposition procedure. Ultrasounds generated a pressure gradient when they entered in this concave end and, therefore, propelled the nanosystem even in high-ionic environments, which is a great advantage in comparison with the systems propelled by hydrogen oxide decomposition, that are only suitable for media of low ionic strengths. Another advantage of ultrasound-based propulsion is that the average rate of the motors is not affected by the functionalization of the gold rods, as is the case with chemically propelled nanowires. Not only complete bacteria but also spores have been captured using micromotors. This is particularly important because spores are much more resistant than bacteria to common chemical antibacterial treatments such as bleach, chlorine dioxide, or paraformaldehyde, as a consequence of their strong protective coating. Thus, polypyrrole (PPy)-COOH/PEDOT/Ni/Pt microtubes functionalized with antibodies against spores of *Bacillus globigii* (*B. globigii*), a simulant of *Bacillus anthracis*, were able to capture these spores from real samples of tap and lake water and then destroy them in a more effective manner, in combination with antimicrobial agents, thanks to the improved mixing caused by the motor motion [47]. 

Circulating tumoral cells (CTC) are the pioneers of the metastatic process and their number in blood is closely related with the disease state and its progression. Therefore, their premature detection is of paramount importance in cancer therapy. Unfortunately, the number of these cells is very low, being 0–10 CTC per mL which is insignificant in comparison with the high number of blood cells (>10^9^ erythrocytes and >10^6^ leucocytes, among others) [48]. There are different methods for their analysis in blood samples reported in the scientific literature, such as the use of magnetic beads functionalized with targeting moieties able to recognize these malignant cells [49] or microfluidic procedures [50]. However, these methods require tedious procedures or extensive sample preparations. As in the previous case, micromotors can facilitate the capture and detection process of these elusive cells. Thus, anti-carcinoembryonic antigen (anti-CEA) monoclonal antibody was anchored on the surface of a tubular microrocket because this antigen is overexpressed in around 95% of colorectal, gastric, and pancreatic cancers [51]. The microrocket presented a ferromagnetic iron layer which allowed its orientation and an inner Pt surface which propelled the system in the presence of H_2_O_2_. These systems were capable of capturing CTCs in PBS and diluted human serum solutions in a selective manner in the presence of cells which did not express the CEA antigen on their surface. Importantly, CTC remained viable during the capture and transport process and after 1 h of exposure to H_2_O_2_ solution at 2% of concentration, which supported the use of these motors for the isolation and subsequent analysis of CTC. Khandare et al. have reported the use of carbon nanotubes with iron oxide nanoparticles in the inner space, and decorated with transferrin on the external surface, for the capture of CTC which overexpress transferrin receptors [52]. Finally, another interesting alternative, different to the cell capture, is to combine the motion capacity of the living organisms with micromotors in order to create hybrid devices able to carry out important tasks, such as controlled fertilization. Thus, Schmidt et al. have trapped bovine sperm cells in the inner cavity of microtubular motors in order to exploit the flagella-based propulsion mechanism of these cells to propel the motors [53,54]. Additionally, immotile sperm cells have been captured by magnetically driven microhelices in order to introduced them into the oocyte for fertilization [55].

## 6. Conclusions

Since the visionary speech of Richard Feynman, scientists of very different fields have struggled with the exotic physical laws of the nanoscale in order to produce tiny machines capable of performing autonomous, controlled and directed motion at the same time that they execute complex tasks such as drug delivery, pathogen, or biomolecule detection and cell transportation, among others. The main advantage of the use of nanomotors for biomolecules or cell detection is their capacity to perform the detection, capture, and transportation process by themselves, without the necessity of carrying out any purification steps. The self-propelled behavior of these motors, in combination with their capacity to be externally guided with the application of magnetic fields, light, or US provide an enormously valuable tool for clinical diagnosis. Despite the great advances carried out in this field in recent years, there is still work to be done in order to improve their abilities. The fabrication methods of these motors should be studied in more detail in order to fabricate even tinier machines (in the sub-micrometer scale) which would allow their use inside the organism and even within cells. It is necessary to develop more biocompatible materials as motor components, because many of the systems which have been reported until now employ toxic metals and non-degradable components, which strongly limit their application in the clinical field. Fuels other than H_2_O_2_ should be applied in order to exploit the natural molecules from which the living systems extract energy. The nanomotors described throughout this review have been employed in vitro or in acellular media. The in vivo application of these nanomotors should be studied in the coming years, which will provide more knowledge about the real impact and liabilities of these systems in the clinical field. This promising field is still in its infancy, but the great potential inside the nanoscale and the inspiring work developed by the nanomotor’s pioneers allows us to have positive expectations about the bright future of these nanometric motors.
